# Investigating cell death mechanisms in amyotrophic lateral sclerosis using transcriptomics

**DOI:** 10.3389/fncel.2013.00259

**Published:** 2013-12-17

**Authors:** Paul R. Heath, Janine Kirby, Pamela J. Shaw

**Affiliations:** Sheffield Institute for Translational Neuroscience, University of SheffieldSheffield, UK

**Keywords:** transcriptomics, cell death, amyotrophic lateral sclerosis, microarray

## Abstract

Amyotrophic lateral sclerosis (ALS) is a motor neuron disease characterized by degeneration and loss of upper and lower motor neurons from the motor cortex, brainstem and spinal cord although evidence is suggesting that there is further involvement of other cell types in the surrounding tissue. Transcriptomic analysis by gene expression profiling using microarray technology has enabled the determination of patterns of cell death in the degenerating tissues. This work has examined gene expression at the level of the tissue and individual cell types in both sporadic and familial forms of the disease. In addition, further studies have examined the differential vulnerability of neuronal cells in different regions of the central nervous system. Model systems have also provided further information to help unravel the mechanisms that lead to death of the motor neurons in disease and also provided novel insights. In this review we shall describe the methods that have been used in these investigations and describe how they have contributed to our knowledge of the cell death mechanisms in ALS.

## Introduction

Amyotrophic lateral sclerosis (ALS) is described as a degenerative disease involving loss of the upper and lower motor neurons from motor cortex, brain stem and spinal cord leading to muscle denervation, wasting and death. There is now increasing evidence that other cell types are involved and the precise pattern of degeneration is unclear (Ince et al., 2011). Multiple mutually compatible mechanisms have been implicated in the pathogenesis of the disease including oxidative stress, excitotoxicity, ubiquitin and proteasome dysfunction, inflammatory activation, RNA processing dysregulation, mitochondrial dysfunction, cytoskeletal abnormalities and activation of apoptosis (Ferraiuolo et al., 2011). However, there remain many unanswered questions as to the origins of the disease and the precise pathogenetic mechanism. The use of microarray technology to explore gene expression at a particular time point and in particular cells is enabling us to unpick some of the specific changes undergone by cells or tissues involved in the disease and also to determine what processes act to protect cells from degeneration. The technology for different array types has been described and reviewed previously and we will not at this time extend this (Clark et al., 2007; Kirby et al., 2007). Whilst there are inconsistencies between the findings of transcriptomic studies and the precise biological mechanisms involved in a disease situation there is no doubt that properly designed studies which generate functionally verifiable results can be invaluable in assisting in the description of the disease process (Bolstad et al., 2004; Rosenfeld, 2010; Henriques and Gonzalez De Aguilar, 2011). For the purposes of this review we concentrated our attention upon articles that were found from a PubMed search using the search terms microarray and ALS.

## RNA quality

A recurring dilemma in studies of gene expression in post mortem tissue has been the residual debate concerning the quality of RNA derived from such tissue and whether it is appropriate for these studies. However, it is now accepted that gene expression studies in post mortem tissue are possible as long as appropriate quality control measures are followed (Harrison et al., 1995; Trabzuni et al., 2011). For most model studies standardized procedures can be set in place in order to reduce potential factors that might cause RNA degradation but even in an established and well organized human brain banking situation there are variables relating to post mortem delay, agonal state and cause of death all of which can affect RNA quality (Tomita et al., 2004; Popova et al., 2008; Durrenberger et al., 2009; Trabzuni et al., 2011). As indicated a number of studies have examined the effect of multiple potential RNA degrading features but the overall consensus is that providing adequate care is taken and good experimental design is incorporated in order to eliminate gross differences between the material being used then a reliable study can be carried out. In order to monitor these potential confounding factors it is important to control for any RNase introduction by carrying out all experimental work in a clean and tidy environment using RNase free materials. In addition it is essential that quality control steps should be included to measure both quantity and quality of the RNA being extracted and used (Copois et al., 2007; Wilkes et al., 2010; Trabzuni et al., 2011). Spectrophotometric methods can be used to measure nucleic acid quantity by examining absorbance at 260 nm. This can give an indication of RNA quality and the 260/280 ratio provides a measure of protein contamination whilst the 260/230 ratio provides a measure of organic solvent carryover. However, these metrics do not give a clear idea of RNA quality. For this, electrophoretic methods are required to examine the RNA directly. In the past a denaturing agarose gel (including formamide in the gel matrix) allowed the RNA to be visualized following staining with ethidium bromide. However, these methods required a large input of total RNA and have now been largely superseded by capillary based methodology such as the Agilent Bioanalyser and/or the TapeStation. In effect, both these devices drive the RNA sample through a capillary and over a laser which monitors absorbance at 260 nm over time hence providing a profile of the absorbance which can be compared to the profile of a molecular weight ladder run at the same time. If total RNA is the test sample then one would expect a small series of peaks at low molecular weight followed by the two ribosomal RNA peaks (the smaller peaks might be removed if a column based separation method is used). Software integral to the machine is then able to calculate an RNA integrity number (RIN) which is scaled from 0 to 10 and depends upon the ratio of the 28S to 18S rRNA peaks and the amount of degraded material that is present. It should be expected that for material derived from tissue culture experiments that a high RIN value is routinely achieved. However, post mortem human material is often quite degraded and RIN values of around 4 might be measured. It has been demonstrated in microarray, RNASeq and qRT-PCR that these values do not preclude a valid experiment (Preece et al., 2003). However, it is important to maintain equivalence in the experimental material and endeavor to compare samples of similar quality as well as support findings through appropriate validation methodologies.

## Human studies in whole tissue

Microarray studies on whole tissue generate an overview of the gene expression profile of the whole tissue at the time of the RNA collection. There have been a number of microarray studies which were designed to examine some aspect of gene expression changes in ALS by examining a particular tissue. A very early study by Malaspina et al. (2001), used gridded cDNA membrane arrays to examine the expression of 18,400 genes in the anterior horn of lumbar spinal cord from four ALS cases and four controls (with a further four patients and controls used as verification subjects). Fourteen transcripts were identified as being significantly differentially expressed, and these were shown to belong to processes relevant to ALS including; oxidative stress and neuro-protection, motor neuron function, lipid metabolism, neuroinflammation and anti-apoptotic factors. This was an important demonstration of the potential of this sort of study and whilst it depended upon a predefined group of cDNAs that were present upon the array, the results gave an overview of gene expression in the anterior horn of the lumbar spinal cord. It also confirmed that post mortem tissue could be used in such a study. Ishigaki et al. (2002) took a different approach. Using lumbar spinal cord from 8 sporadic ALS cases and controls they initially used one case and control for molecular indexing which identified 576 cDNA fragments in the sALS material that could be compared to the control to select identifiers for further analysis. In this way spotted microarrays were prepared for 84 cDNAs deemed to be differentially expressed between the sALS and control individuals that were further examined on a wider group of patients and controls. Ultimately six differentially expressed genes were identified; four corresponded to previously known genes, 30-kDa TATA-binding protein-associated factor (*TAFII30*), macrophage-inhibiting factor-related protein-8 (*MRP8*), metallothionein-3 (*MT-3*) and ubiquitin-like protein 5 (*UBL5*) whilst the remaining two were uncharacterized expressed sequence tags. This was an interesting approach but the limited numbers of genes identified made it difficult to identify processes related to cell death.

An early study by Dangond et al. (2004), used Affymetrix FL arrays to compare the expression profile of grey matter from the lumbar spinal cord of seven ALS cases and four controls. A robust analytical process identified 93 genes which discriminated between the two groups. There was some attempt to differentiate between sporadic and familial disease, however, the numbers in the familial group made this analysis less robust. The 93 significant genes were subdivided into groupings defined by known biological functions giving some 10 sub-groups including; transcription and RNA binding, excitotoxicity, cell survival and growth, neuronal function, inflammation, cell receptors and signaling and stress response. Wang et al. (2006), examined the motor cortex in five ALS cases compared to three controls using the newer Affymetrix U133A Gene Chips containing 14,500 genes for microarray analysis. They identified 275 significantly differentially expressed genes, of which 265 were downregulated and they were able to verify the changes in 22 of these genes by quantitative real time polymerase chain reaction (QRT-PCR). Interestingly different groups of genes seemed to be important in sALS motor cortex compared to the spinal cord, but there were similarities in that stress response and excitoxicity were featured in both groups, as were features of mitochondrial metabolism. Unfortunately, the different cell populations of the two regions might be having a profound impact upon the data. A further study of motor cortex (Lederer et al., 2007), used 11 sALS cases and 9 controls in an Agilent slide array based study. Fifty-seven genes were identified as differentially expressed following the filtering and pathway based analysis; 40 down and 17 upregulated. Such a small group of genes were readily verifiable by alternative methods and the list was compared to previous studies including that of Dangond et al. (2004), and 14 genes showed a similar response. Overall a very thorough analysis produced an interesting interacting pathway diagram examining the interplay of differentially expressed genes in the motor cortex of sALS individuals. It is important to note that without describing individual genes it is possible to discern similar patterns in the lists of genes differentially expressed in the whole tissue experiments described here. Recurrent themes are mitochondrial metabolism, excitotoxicity, calcium homeostasis, protein turnover and neuronal maintenance and signaling.

Using a different approach Offen et al. (2009) examined the gene expression profile in spinal cord of sALS patients and compared some of the changes to the Glycine 93 to Alanine (G93A) SOD1 mouse model of fALS. In the study of four sALS cases compared to four matched controls using the Clontech membrane arrays, 60 gene changes were identified in the 1176 probe sets available to be examined. On this occasion the genes identified were grouped into biological processes such as; apoptosis, protein turnover, cell cycle functions, extracellular carrier/transport functions, cytoskeletal and intracellular proteins and proteins relating to transcription and translation. Six genes were further investigated in the G93A *SOD1* mouse model of fALS to determine if the changes seen post mortem were recapitulated during the lifespan of the mouse. These genes were; cathepsins *B* and *D*, apolipoprotein *E*, epidermal growth factor receptor (*EFGR)*, ferritin and lysosomal trafficking regulator (*LYST)*. These transcripts did seem to increase with disease progression in the mouse model suggesting some overlap between the human situation and the mouse model which does add some credence to the use of the model for human ALS.

Two recent studies have taken a quite different approach. Bernardini et al. (2013), concentrated upon mitochondrial related genes and Figueroa-Romero et al. (2012), tried to relate gene expression changes to methylation events in the genome. The Figueroa-Romero study was focussed on relating genome methylation events to gene expression changes found in sALS spinal cord. 12 sALS cases and 11 age and sex matched controls were used to determine the methylated genetic regions using Illumina Methylation 27 Bead Chips, whilst gene expression analysis was carried out using the Affymetrix Human U133 Plus 2 GeneChips. In the methylation chip assay 3574 regions associated with genes were found to be differentially regulated. In the gene expression study some 1182 genes were found to be differentially expressed in the sALS spinal cord. When comparing the two datasets 51 genes that were hypomethylated were upregulated and 61 genes that were hypermethylated were downregulated. These concordant “epigenes” could be functionally grouped into categories such as; immune response, defence response, neuron adhesion and plasma membrane. Overall this work suggested an epigenetic, or environmental, influence upon the pathogenesis of sALS which may be relevant to the process of cell survival. In the Bernardini study they concentrated upon skeletal muscle which was a new, but potentially relevant, tissue to examine. They hypothesized that muscle degeneration might be functionally important in the process of neurodegeneration and that differentially expressed genes in this tissue could be key in the pathogenic process. They examined quadriceps or biceps muscles in seven sALS cases and seven age and sex-matched controls. A focussed Affymetrix GeneChip array was used and using a fold change cutoff of 1 and *p*-value cutoff of 0.05, 96 differentially expressed genes were identified. Unlike in previous studies on this occasion more genes (80), were seen in the upregulated group. In total 13 genes related to muscle structure were distinguished and when ontology relationships were investigated using the DAVID software (Huang da et al., 2009) platform, other genes were found to be grouped in areas such as the actin cytoskeleton and glycogenesis, with mitochondrial function also identified. Further studies would be needed to determine the relevance of this work particularly with regard to further subtyping of the ALS cases. A study by Shtilbans et al. (2011) also looked at gene expression differences in muscle biopsies from ALS cases and controls. The study was hampered in that mixed groups of familial and sporadic cases were examined and different muscle groups were included in mixed proportions in each comparative group. The differences they found were in genes related to muscle structure, the cytoskeleton and cellular metabolism.

Two studies have used peripheral blood to look for gene expression differences with the underlying hypothesis that changes found in this tissue might reflect changes associated with the disease as has been seen in other neurodegenerative diseases. Saris et al. (2009) used whole blood and a designed analysis tool to uncover disease specific differences whereas Mougeot et al. (2011) isolated lymphocytes and examined these directly. In the Saris study, the weighted co-expression analysis found that genes in pathways related to apoptosis, the mitochondrion, stress response, calcium binding ubiquitination and vesicle transport were differentially expressed between the ALS patients and controls. In contrast, Mougeot used an ontology relationship tool called SAFE and found that a set of genes previously grouped into an ALS related KEGG pathway were differentially expressed in the lymphocytes. Genes in this pathway related to motor neuron degeneration in ALS (KEGG:05014), and included SOD1, caspases 1,3,9, neurofilament light, medium and heavy (*NFL, NFM* and *NFH)*, and N-methyl-D-Aspartate receptors *GRIN 1, 2A, 2B, 2C* and *2D*. In addition, an ontology analysis based upon individual genes identified changes in pathways related to DNA metabolism, RNA splicing, mitochondrial function, oxidation, endoplasmic reticulum,ubiquitin proteasome system, post-transcriptional modification and neurological function. The overall conclusion to be drawn from both studies in blood is that although it is a peripheral tissue, it does show gene expression changes and pathways previously implicated by the use of central nervous system (CNS) material, including apoptosis, mitochondria, RNA splicing and, surprisingly, neurological function.

The studies of whole tissue have reached different conclusions, perhaps as a result of using tissue from different stages of disease and location, but there are common themes involved in the processes that are found to be differentially expressed in the ALS tissue which have been associated to motor neuronal cell death. In particular these are apoptosis, oxidative stress, protein processing, mitochondrial function and the cytoskeleton.

## Single cell analysis of human motor neurons

The previous studies are valuable but they make it difficult to determine the precise input of each cell. In order to obviate this, several methods have been developed to examine the gene expression profile of individual cells isolated from tissue. Two recent reviews have discussed the available methods for cell specific transcriptome analysis (Kannanayakal and Eberwine, 2005; Okaty et al., 2011), and we shall not discuss these further. In terms of ALS, most studies seem to have adopted a laser capture microdissection approach. In 2005, Jiang et al. carried out a microarray study of laser captured motor neurons from the lumbar region of 14 sporadic ALS cases and 13 controls (Jiang et al., 2005). They used a PALM microdissector and applied the labeled RNA to BD Atlas glass microarrays. They found 52 up and 144 down regulated genes from the 4845 interrogated. Owing to the fact that they compared the isolated spinal motor neurons data to ventral horn spinal cord tissue data they were able to distinguish genes whose differential expression was restricted to the motor neurons and which was not detected when the whole tissue was examined. It was found that genes associated with cell death and cell signaling were upregulated in the disease related motor neurons whereas those associated with the cytoskeleton and transcription were downregulated. This study provided information concerning gene expression changes that were specific to the spinal cord motor neurons and demonstrated the value of this approach over whole tissue studies. Our group has carried out a number of published studies examining the gene expression profile of motor neurons isolated by laser capture microdissection in several models. In her study in 2010, Cox et al. (2010) examined the differences between three individuals who had charged multivesicular body protein 2B (*CHMP2B)* mutations leading to an ALS phenotype and 7 matched normal controls. Following gene expression analysis using Affymetrix Human U133 Plus 2 GeneChips, 890 genes were downregulated and 55 upregulated at a fold change cutoff of 2 and *p*-value 0.05. These were categorized using DAVID software into ontological groupings and groups associated with axon guidance, regulation of actin cytoskeleton and soluble NSF attachment protein receptor (SNARE) interactions in vesicular transport, mammalian target of rapamycin (*mTOR*) signaling and regulation of autophagy, mitogen activated kinase (*MAPK*) signaling, calcium signaling, and cell cycle and apoptosis were found to be the most enriched. Further functional studies were able to confirm a dysfunction in the autophagy pathway in an *in vitro* model. A further study (Kirby et al., 2011), examined gene expression differences in cervical spinal motor neurons between three fALS cases carrying SOD1 mutations and seven normal controls using the Affymetrix Human U133 Plus 2 arrays. In total, 524 probe sets were found to be increased and 646 decreased. These were characterized using the DAVID software package and the major enriched categories were transcription, signaling and metabolism. Importantly further investigations demonstrated the relevance of the cell survival pathway involving *PTEN/AKT* in the motor neurons with anti-apoptotic genes being downregulated in the surviving motor neurons indicating an attempt by these cells to mount a pro-survival response. Comparison of these studies indicates that the different genetic variants have distinct gene expression changes which ultimately lead to motor neuron death.

In contrast to the case control scenario, Brockington et al. (2013), examined features that distinguish the motor neurons from the oculomotor nucleus and the lumbar spinal cord in normal individuals to determine those features that enable the oculomotor motor neurons to be selectively resistant to the cell death undergone by spinal motor neurons in ALS. Tissue from four neurologically normal individuals was collected and laser capture microdissection used to isolate motor neurons from the oculomotor nucleus and lumbar spinal cord. The labeled RNA from these was applied to the Affymetrix Human U133 Plus 2 GeneChip. 1521 gene expression differences were identified as being differentially expressed in the oculomotor neurons. Gene ontology analysis determined that genes involved in synaptic transmission, ubiquitin mediated protein degradation and mitochondrial oxidative phosphorylation were upregulated in oculomotor motor neurons; these pathways had shown decreased expression in the ALS spinal cord and motor cortex in previous studies. This work was supplemented by carrying out comparison studies with other gene expression data derived from an online database and the analysis showed that the differences observed in human oculomotor neurons were also found in two other species confirming that the oculomotor motor neurons had a particular profile of synaptic neurotransmitter receptors, particularly gamma aminobutyric acid (*GABA)* and glutamate which made them less vulnerable to excitotoxic cell death. Electrophysiological studies complemented and supported this conclusion.

Again some common features can be derived that seem to associate the differential expression of genes related to the mechanisms of cell death. These include; cell signaling, autophagy, phosphatase and tensin homologue/protein kinase B (*PTEN/AKT)* cell signaling pathway, ubiquitin and mitochondrial function, cytoskeleton, transcription and apoptosis.

## Animal models using whole tissue

Whilst human tissue can only be accessed at end stage, the use of animal models allows progression of the disease to be monitored. An early study by Yoshihara et al. (2002) examined the gene expression differences between tissue homogenates of lumbar spinal cord of G93A SOD1 versus non-transgenic littermates at three ages; 7, 14 and 17 weeks corresponding to presymptomatic, onset and end stage of disease. Using mouse Atlas arrays from Clontech they found an upregulation of inflammatory related genes associated with activated microglia and astrocytes. This was induced by 11 weeks of age and continued to advance up to the 17 week time point.

Fukada et al. (2007) examined the gene expression profile of whole spinal cord from two time points, presymptomatic (98 days) and post symptomatic (154/176 days), in a different mutant SOD1 transgenic model carrying the L126delTT mutation. They used the AceGeneMouse Oligo Chip. At the presymptomatic stage 11 genes were upregulated and two downregulated at a fold change of greater than two whilst at the post-symptomatic stage 54 were upregulated and four downregulated. With such small numbers of differences ontological relationships were hard to carry out and since whole spinal cord was examined the precise relationship of the gene changes to the disease process is difficult to disentangle.

It is thought that physical exercise might have a beneficial effect upon disease progression in ALS and two studies investigated this using microarrays and mouse models (Ferraiuolo et al., 2009; Hashimoto et al., 2009). Hashimoto et al. (2009) examined the response to exercise in normal mice by subjecting the mice to two exercise regimes; a single burst of 30 min exercise or 2 weeks of 30 min exercise per day on a treadmill. Whole spinal cord RNA was examined using the OpArray Mouse V4 array from Operon. Only a small number of changes in gene expression were identified; after the single burst of exercise 3 genes were upregulated and 29 downregulated whilst following the extended exercise regime 1 gene was found to be increased and 13 decreased. As seen in some previous studies the numbers are rather small for pathway analysis and since whole tissue has been studied it is difficult to dissect out precise effects. Ferraiuolo et al. (2009) examined the gene expression changes in gastrocnemius muscle as well as motor neurons isolated from lumbar spinal cord of three female mice subjected to a voluntary exercise regime of 21 days on a running wheel compared to three sedentary mice. Data were also collected concerning the exercise behavior of the mice. In the muscle tissue, 194 genes were upregulated and 176 downregulated using the same parameters. These genes were functionally found to be associated with vascularization and myogenic processes involved with extracellular matrix reorganization. The profiles exhibited by the gastrocnemius muscle are representative of the mixed cell population of the whole tissue and indicate some overall changes in the muscle as a response to the exercise regime. Overall, 203 genes were upregulated and 241 down at a fold change of at least two and *p*-value of 0.05 or less in the motor neurons. Functionally these genes were categorized into signaling, cytoskeleton and transcription regulation. The response of the motor neurons to exercise was similar to that of hippocampal neurons to repetitive stimulation, akin to a long term potentiation. There is a body of evidence that links exercise to the processes of cell death observed in ALS. This work goes some way to highlight similarities between the physiological response of MNs and skeletal muscle to exercise and the pathophysiology of ALS.

Two recent studies from the same group (Chen et al., 2010; Hu et al., 2013), have used Mouse Exon 1 arrays from Affymetrix to examine gene expression changes and alternative splicing in the G93A SOD1 mouse model. In the first study they examined animals at onset versus litter mate controls and in the second study 30 day transgenic mice were compared to litter mate controls of the same age and 120 day transgenic mice. They examined both differential expression and alternative splicing events. There were only 202 differentially expressed genes with a *p*-value cutoff of 0.05 found when comparing the 30 day old transgenic to nontransgenic mice but only one of these was at a fold change of > 2. There were in total 2869 differentially expressed genes events found when comparing the 120–30 day transgenic mice with 263 up and 71 down when a fold change of two and *p*-value of < 0.05 was imposed. Gene ontology analysis of the differentially expressed genes revealed that pathways involving cytokine receptor, cell adhesion, haematopoesis, and cell signaling included the most genes. In the onset group 322 transcripts were differentially expressed with 309 upregulated in the transgenic group when of a fold change of two and *p* < 0.05 was applied. Similar pathways were identified in this study to the previous one. In both studies a splicing index analysis was carried out in order to determine the levels of alternatively spliced transcripts. Comparing the 120 day transgenic to 30 day control 563 transcripts were alternatively spliced, of which 537 were considered as exon inclusion events, whilst only 85 alternative splicing events were identified in the 30 transgenic versus 30 day control comparison and on this occasion 61 were classified as exon inclusion. When the splicing data was examined by pathway analysis it was interesting that similar pathways were highlighted as found in the differentially expressed genes analysis. The data was interpreted to demonstrate that as the disease progressed then an increased number of splicing changes were seen. To some extent this is borne out by the onset study where 333 probe sets showed an altered splicing index indicating possible alternative splicing. However, the comparison of 120 day transgenic mice versus controls is missing which would allow a better comparison relative to normal ageing effects. Whilst some qRTPCR validation was completed the studies represent early work in using the exon arrays to examine the changes invoked by the presence of the G93A *SOD1* mutation on alternative splicing events.

The studies discussed thus far have used mouse models but Hedlund et al. (2010) used the G93A SOD1 rat model for a study of selective motor neuron vulnerability. They quantified the extent of motor neuron loss in different motor neuron nuclei; oculomotor and trochlear (CN3/4), facial (CN7), trigeminal (CN5), hypoglossal (CN12) and cervical spinal cord in the transgenic model. This directed the laser capture studies where motor neurons were collected from the CN3/4, CN12 and cervical spinal cord of normal rats and applied the isolated RNA to whole genome rat microarrays (Rat 230 2 Affymetrix). The microarray analysis showed that the more vulnerable motor neurons of the cervical spinal cord and hypoglossal nerve were more similar than either group compared to the less vulnerable CN3/4 motor neurons. There were interesting differences in Hox gene expression between the motor neuronal groups which were concomitant to the relative positions of the neurons in the anterior to posterior axis. There were also differences in RNA processing between the groups which also seemed to relate to the known association of RNA processing genes with ALS (Baumer et al., 2010). This report highlighted the usefulness of alternative animal models for the study of ALS and took an interesting approach to uncovering some of the underlying differences between selectively vulnerable and resistant motor neurons.

Dupuis and Loeffler (2009) examined neuromuscular changes in transgenic models of ALS. This led from the observation that the first event in the disease process seen in the transgenic mouse model of ALS is the destruction of the neuromuscular junction. Hence, studies concentrating upon the motor neurons may be identifying effects rather than cause of disease. However, it must be borne in mind that the SOD1 mouse model represents an overexpression model of disease and that SOD1 mutations correspond to only 2% of all ALS.

Finally in this section, Kumimoto et al. (2013) have undertaken to study ALS in a Drosophila model. They used a GAL4-UAS promoter to drive expression of wild type Drosophila SOD1 or the human SOD1 mutation glycine 85 to arginine (G85R) in motor neurons and glia. They carried out microarray analysis upon whole flies 5 days old (young), and 45 days (old), using the Drosophila 2 GeneChips from Affymetrix. They used several analyses to examine gene expression changes at the different ages and in the different cell types as well as a meta-analysis to uncover general changes. The mutant *SOD1* G85R in motor neurons alone caused 58 gene expression changes (33 up) at 5 days and 102 transcripts (73 up) at 45 days whereas in glia alone these numbers were 65 (33 up) and 105 (57 up) respectively. When the transgene was expressed in both cell types simultaneously the numbers were; 70 (34 up), and 83 (38 up). The overexpression of G85R SOD1 in the cells of the Drosophila caused changes in gene expression in oxidative stress, mitochondria, lipid metabolism and neurodevelopmental and signaling genes. Since the microarray analysis was carried out upon whole flies it is again difficult to determine the precise cellular effect of the overexpression strategy but there were commonalities in the pathways implicated with previous microarray studies and the impression is that the study offers a new model approach to understanding ALS. Since these studies were somewhat varied in the animal models, the tissue being investigated and the number of genetic changes identified it is difficult to describe common themes in the gene expression changes being seen and their relationship to cell death. However, cell signaling, mitochondrial dysfunction and oxidative stress continue to be implicated in the pathogenesis of ALS in animal models.

## Single cell analysis of non-human model motor neurons

Perrin et al. (2005) combined laser capture dissection with microarray to examine gene expression in the spinal cord motor neurons of the G93A *SOD1* mice. Lumbar spinal motor neurons from male mice at presymptomatic (60 days), onset (90 days) and end stage of disease (120 days) were analyzed using the Mouse 430 2 GeneChip from Affymetrix. At 60 days only 27 genes were differentially expressed with this number increasing to 150 at 90 days and more than 400 by 120 days. At all the time points more genes were found to be upregulated in the G93A SOD1 mice; 17, 95 and 389 respectively. Thirteen of these transcripts were found to be differentially expressed at all stages. A similar study from our group (Ferraiuolo et al., 2007), looked at the effect of both the G93A SOD1 and the wild type SOD1 compared to non-transgenic littermate controls on a homogeneous background of C57bl6. Few changes were seen in motor neurons isolated from the WTSOD1 mice but 252 genes were differentially expressed at 60 days (234 upregulated), 51 at 90 days (32 up) and 167 at 120 days (81 up), in the G93A SOD1 mice. These changes at the presymptomatic, symptomatic and end stage of the disease process in the mice indicate that at an early time point the motor neurons try to mount a metabolic response to the burgeoning disease process by upregulating several pathways. However, by the endstage, this process having failed, the cells appear to try to re-enter the cell cycle which is a doomed response. There are notable differences between the two studies in terms of the overall changes in gene expression profile, although both groups do see changes in the intermediate filament protein vimentin, it is likely that the differences might relate to the actual genetic background of the mice being used, the transgene copy number and also the analytical processes used in examining the data. A study by Saxena et al. (2009) took a different approach to examine the matter of selective vulnerability of motor neurons. They injected RITC-dextran into the gastrocnemius or soleus muscles and following retrograde transport they were able to identify and selectively isolate the motor neurons associated with the vulnerable phenotype which project to the fast fibre gastrocnemius muscle and those that are relatively resistant and project to the soleus muscle which has fatigue resistant or slow fibres. The RNA isolated from the selectively microdissected motor neurons was applied to the Mouse 430 2 arrays from Affymetrix. The longitudinal study showed that the vulnerable motor neurons are selectively prone to endoplasmic reticulum stress and a progressive failure to mount a proper unfolded protein clearance response.

This group of studies are more focussed and underline the importance of the single cell analysis at different stages of disease progression.

## Single cell analysis of non-human non-motor neuron cell types

Using the G93A Mutant SOD1 mouse model Ferraiuolo et al. (2011) attempted to examine the relationship between motor neurons and astrocytes in this model of disease (Ferraiuolo et al., 2011). Astrocytes were collected from lumbar spinal cord of three male 60 day old mice and three non transgenic litter mate controls, and the labeled RNA applied to Mouse 430 2 Gene Chips from Affymetrix. 583 transcripts were found to be upregulated 526 downregulated in the G93A SOD1 astrocytes. Ontological analysis showed that multiple transcripts associated with carbohydrate metabolism were differentially expressed and an overall decline in the activity of the lactate shuttle was described in the mutant astrocytes indicating an inability to support the metabolic requirements of the motor neurons. The altered metabolism was further investigated and it was shown that non-transgenic motor neurons grown on G93A SOD1 astrocytes demonstrated an increase in the ratio between pro- nerve growth factor and mature nerve growth factor (NGF) which was associated with an over-expression of the p75 receptor for NGF. Hence it was shown that the G93A SOD1astrocytes provided reduced metabolic support to the motor neurons as a result of the downregulation of the lactate shuttle and the activation of the p75 receptor and these factors contributed to the toxicity of the mutant astrocytes to the co-cultured motor neurons.

## Cell Culture model studies

Two studies have examined the gene expression profile of the NSC 34 cells carrying different human SOD1 mutations. The first study (Kirby et al., 2002), utilized a designed membrane microarray from Clontech with cDNAs for just 588 genes. The experiment was carried out on cells carrying G93A, G85R or isoleucine 113 to threonine (I113T) SOD1 mutations at basal conditions and cells which had undergone stress exerted by serum withdrawal. At the basal condition differences were seen in the numbers of differentially expressed genes for the different SOD1 mutation but a combined analysis showed only 29 genes to be differentially expressed as a result of the SOD1 mutation with seven being downregulated. Very few genes were found to be differentially expressed following the induction of a stress imposed by serum withdrawal which might be a result of the analytical stringency being applied. In a subsequent study (Kirby et al., 2005), a comparison was made between NSC34 cells expressing G93A mutant SOD1 and those expressing wild type human SOD1. On this occasion the mouse U74Av2A GeneChip from Affymetrix was used. At a fold change of two with a *p*-value of 0.05, 268 transcripts were differentially regulated in the presence of mutant SOD1, of which 197 were downregulated. Following thorough ontological analysis it was found that the presence of the mutant SOD1 caused a significant down-regulation of the cells capacity to deal with oxidative stress. In particular the group of genes with an antioxidant response element in their promoter, termed programmed cell life genes, which would be activated by the transcription factor nuclear factor (erythroid-derived) like 2 *(Nrf2)* were downregulated indicating a failure of the cells to mount a proper therapeutic antioxidant stress response. Subsequent work by Mead et al. (2013) has demonstrated that induction of the *Nrf2* response by S(+)-Apomorphine improves the motor function of the G93A *SOD1 *mice underlining the importance of increasing the expression of the programmed cell life genes in a therapeutic strategy to treat ALS.

Vargas et al. (2008) isolated primary astrocytes from both G93A SOD1 mutant and normal littermate control embryonic rats and examined the profile of gene expression using Rat genome 230 2 arrays from Affymetrix. A total of 81 transcripts were differentially expressed by the mutant SOD1 bearing astrocytes with 55 being upregulated. The genes were characterized according to their biological function and were separated into groups; extracellular matrix and cell migration, signaling and receptor activity, transcription, cell proliferation, response to stress, oxidoreductase activity, catalytic activity and unknown. It is interesting to note that despite the fact that different cell types are being investigated there are some similarities in the effect of the *SOD1* mutation in astrocytes and motor neurons from mice and rats.

In the study by Boutahar et al. (2011) they derived primary cortical motor neurons from transgenic mice either with or lacking the G93A *SOD1* mutation. The cells were derived from E14 embryos and maintained in culture for a brief period until the presence of the transgene was confirmed. Some cultures were subjected to stress with hydrogen peroxide or NMDA treatment. Microarrays using Mouse 430 2 GeneChips from Affymetrix were carried out and the results analyzed to determine fold changes of two or more. In the untreated cells 260 transcripts were differentially regulated with 70 upregulated. Following hydrogen peroxide treatment the number was 163 with 59 upregulated and following NMDA treatment 181 with 62 upregulated. Few genes were shared between the three treatments. Gene ontological analysis showed an increase in transcripts associated with the proteasome, some associated with autophagy and cytoskeletal organization and axonal transport. There was some downregulation in transcripts associated with ion transport. It was noted that the presence of the transgene alone increased cell death but this process was mitigated when the additional stresses, hydrogen peroxide or NMDA, were introduced. Hence they identified a small group of genes which were upregulated in the untreated G93A cells but downregulated following the introduction of an additional stress. These genes were; Cathepsin H,(*CSTH*) and Autophagy related 4 homolog D (*ATG4D*) which are involved in protein degradation, tubulin beta-4B chain (*TUBB2C*) and Rho guanine nucleotide exchange factor 11 (*ARHGEF11*) which are cytoskeletal, cell division cycle 25 homolog (*CDC25*) which is involved in cell cycle regulation, solute carrier 7 (*SLC7A12*), DEAD box 43 (*DDX43*) and leucine rich recognition motif 4 (*LRRTM4*) which are involved in the transmembrane domain and transcripts *ACN9, SSBP9* and *TRIM36* involved in transcription regulation.

The studies in cellular culture models all seem to underline the importance of oxidative stress in the pathogenesis of ALS and the difficulty that cells containing mutant SOD1 have in dealing with this metabolic insult.

## Systems Biology

Some studies have taken a more broad brush approach to the analysis of microarray data and attempted to incorporate data from a number of sources. Kudo et al. (2010) developed an integrative approach to examine post mortem human material, mouse models and microarray methods. They used the mouse models of ALS, G93A SOD1 and frontotemporal dementia (FTD), P30L Tau and isolated both motor neurons and glia for microarray analysis. Using Agilent whole mouse genome microarrays 251 transcripts were identified as being differentially expressed in at least one of the four comparisons. Of these 186 corresponded to known genes and ontological analysis revealed that the most enriched biological processes were associated with protein modification/phosphorylation, signaling, regulation of muscle contraction, stress response, the immune system and cell communication. The data were confirmed both in further murine tissue by semi-quantitative PCR and in human tissue from sALS cases with tissue microarrays. Hence they used complementary methodology to identify common mechanisms in the SOD1 and TAU mouse models of motor neuron degeneration and human sALS. The genes *CNGA3, CRB1* and *OTUB2* were common to all the models and *CNGA3, CRB1, OTUB2, SLK, DDX58, RSPO2* and *MMP14* were related to sALS. In addition, 13 transcripts found differentially regulated in the SOD1 motor neurons were also found to be altered in their expression in the blood of G93A SOD1 mice.

Baciu et al. (2012) adopted a reanalysis approach where they performed an in depth analysis of a microarray experiment carried out using purified peripheral blood lymphocytes from 11 ALS patients and 11 age matched controls. The RNA had been interrogated using Agilent 4 × 44k human arrays and the authors used a modified BaFL pipeline and at the same time a severe TM4 statistical analysis to remove potential confounding probes sets and “purify” the dataset. The methodology was validated using a previous dataset carried out using similar microarrays and a coronary heart disease study. By carrying out the two different analytical methods the authors attempted to account for errors that might be inherent to either alone. Ultimately, a series of seven genes was identified as being upregulated in the sALS patients, were common to both methods and were verifiable by qRTPCR. These genes were; *B2M, ACTG1, DYNLT1, SKIV2L2, C12orf35, TARDBP* and *ILKAP* (which cross-hybridises with TARDBP).

A recent meta-analysis from Saris et al. (2013) looked to examine the datasets from a number of different microarray studies of both murine and human material. They included seven human studies; three of lumbar cord, two cervical cord and two motor cortex. In addition there were nine studies of transgenic mouse model material; seven of G93A SOD1, one L126delTT SOD1 and one study of the progressive motor neuropathy and wobbler mice. As the authors conclude, despite the variety of input material and different array platforms used, there was a level of consistency in the altered gene expression output. Hence they found altered gene expression in the areas of protein turnover, immune response and apoptosis in both human and murine studies. The mouse studies also showed consistent differences in genes involved in the lysosome, metal ion binding and mitochondrial function. This study is an interesting new approach to attempt to summate all the data that has been produced thus far. There are likely to be further assays of this type which will take advantage of the large datasets in order to generate informative data with greater statistical power owing to the larger numbers of samples being included.

## Concluding remarks

This short review has attempted to summarize the depth of study that has been carried out using transcriptomic technology to examine the gene expression changes that can be identified in comparisons of ALS tissue and controls. With the variety of studies that have been done it is quite difficult to accurately summarize the information but some overall differences can be seen. In terms of mechanisms that have been uncovered in these studies that are influential in the processes that underlie cell death in ALS it can be seen that certain pathways are repeatedly enriched as a consequence of the disease process. These include; oxidative stress, mitochondrial function, apoptosis, cytoskeleton, neuroinflammation and protein processing. It is interesting to note that when peripheral tissues have been used similar processes have been identified as distinguishing the material from individuals with disease. Whilst many of the studies delineated differences between disease and control at the level of the transcriptome an important development has been developing functional studies to demonstrate the validity of the identified changes and then subsequent studies to investigate mechanisms to ameliorate them.

It is likely that the use of the microarray technology is likely to become more of an adjunct to support a body of work as seen in the study from Egawa et al. (2012). In this case the transcriptomic element of the work was used to characterize human induced pluripotent stem cells derived from dermal fibroblasts of normal individuals and several TDP43 mutants. In the past we have seen the transcriptomic study as being the lead in the experimental approach but on this occasion the development of the induced stem cells was the primary goal and the microarray analysis was simply a tool to assist in the characterization of derived cells and define potential routes for drug intervention. It is likely that with the maturity and confidence that several years of transcriptomic research have generated this type of approach will become more widespread.

A further development in the examination of gene expression differences between tissues is going to be the use of next generation sequencing to examination gene expression directly rather than as an indirect measure based upon manufactured probes. The next generation sequencing methodology provides a more detailed examination of the transcriptome which allows for a more in depth examination of novel transcripts, splice junctions and non-coding RNAs in a single step rather than having to carry out multiple different array experiments. As with the early microarray work there is not yet a defined analytical pathway for interpreting the data from RNA Seq experiments but this is progressing rapidly. There is evidence that the two tools are being used in a combinatorial approach (Kogenaru et al., 2012).

Transcriptomics has been used successfully to investigate the mechanisms underlying the processes of cell death and has provided insights into the pathways that may be dysregulated. As the tools are becoming more established it is likely that more studies will use them to assist in the functional studies needed to develop new treatments for ALS.

## Author contributions

Paul R. Heath devised and produced the manuscript, Janine Kirby and Pamela J. Shaw contributed editing and revising of the manuscript.

## Conflict of interest statement

The authors declare that the research was conducted in the absence of any commercial or financial relationships that could be construed as a potential conflict of interest.
